# The influencing factors of infectious complications after percutaneous nephrolithotomy: a systematic review and meta-analysis

**DOI:** 10.1007/s00240-022-01376-5

**Published:** 2022-12-14

**Authors:** Guiming Zhou, Yuan Zhou, Rui Chen, Daoqi Wang, Shumin Zhou, Jiao Zhong, Yuan Zhao, Chuanping Wan, Bin Yang, Jinming Xu, Erkang Geng, Guoxiong Li, Yunfeng Huang, Haoran Liu, Jianhe Liu

**Affiliations:** 1 Department of Urology, The Second Affiliated Hospital of Kunming Medical University, NO. 374 Dianmian Avenue, Wuhua District, Kunming, China; 2 Department of Urology, The First Affiliated Hospital of Anhui Medical University, Hefei, China; 3 Xishuangbanna Dai Autonomous Prefecture People’s Hospital, Jinghong, Xishuangbanna, Yunnan China; 4 Kunming University of Science and Technology, Kunming, Yunnan China; 5 Menghai County People’s Hospital, Menghai, Xishuangbanna, Yunnan China

**Keywords:** Influence factors, Infectious complications, Percutaneous nephrolithotomy, Sepsis, Systemic inflammatory response syndrome, Urinary tract infection

## Abstract

Infection is the most common complications of percutaneous nephrolithotomy (PCNL) in treating urinary calculi. However, the risk factors for developing infectious complications after surgery have not been clarified, and the predictive value of some factors is controversial. This study aimed to assess the risk factors for postoperative infectious complications of PCNL. We performed a systematic search of PubMed, Web of Science, Cochrane Library, and EMBASE to obtain studies reporting risk factors for postoperative infection complications after PCNL. In this review, demographic factors, laboratory test factors, and perioperative factors were evaluated. The odds ratio (OR) or mean difference (MD) with a 95% confidence interval (CI) was calculated to assess the risk factors. A total of 18 studies were included, with a total of 7161 study patients with a mean age of 46.4 to 55.5 years and an incidence of infectious complications after PCNL ranging from 2.4% to 40.4%. Twelve factors were identified as independent risk factors for post-PCNL infection complications (*P* < 0.05), female (OR = 1.60, 95% CI 1.23–2.07), positive urine culture (UC) (OR = 3.16, 95% CI 2.11–4.74), positive renal pelvis urine culture (RPUC) (OR = 5.81, 95% CI 1.75–19.32), positive stone culture (SC) (OR = 5.11, 95% CI 1.46–17.89), positive urine leukocyte (OR = 3.61, 95% CI 2.45–5.34), infected stones (OR = 7.00, 95% CI 1.27–38.55), elevated blood leukocyte (MD = 0.71, 95% CI 0.31–1.10), elevated neutrophil-to-lymphocyte ratio (NLR) (MD = 0.55, 95% CI 0.43–0.66), preoperative stenting (OR = 1.55, 95% CI 1.10–2.20), multiple puncture access (OR = 2.58, 95% CI 1.75–3.82), prolonged operative time (MD = 10 20, 95% CI 4.80–15.60), and postoperative residual stone (OR = 1.56, 95% CI 1.24–1.98). Female, UC positivity, RPUC positivity, SC positivity, urine leukocyte positivity, infected stones, elevated peripheral blood leukocytes, elevated NLR, preoperative stent implantation, multiple puncture channels, prolonged operation time, and postoperative residual stones were identified as independent risk factors for infection complications after PCNL.

## Introduction

Percutaneous nephrolithotomy (PCNL) is a surgical technique with less trauma, more efficient stone extraction, less postoperative pain, less bleeding, and faster recovery than open and laparoscopic surgery and is now a common surgical method for the treatment of upper urinary tract stones [1]. However, compared with extracorporeal shock wave lithotripsy, and retrograde ureteral flexible lithotripsy, PCNL is still the most invasive and complicated procedure for treating upper urinary tract stones [2].

Infection is the most common complication after PCNL, and studies have shown that the incidence of systemic inflammatory response syndrome (SIRS) is as high as 35% in patients with complex stones [3]. Without effective early intervention, sepsis can develop further, with an incidence of 0.3 – 7.6%, and sepsis has been reported to be the most common cause of death in patients after PCNL [2, 4, 5]. Therefore, a comprehensive evaluation of the factors associated with post-PCNL infectious complications should be performed. However, the risk factors for developing infectious complications after surgery have not been clarified, and the predictive value of some factors is controversial [6–9]. For this reason, this paper conducts a systematic evaluation and meta-analysis from three aspects, demographic factors, laboratory factors, and perioperative factors, to comprehensively explore the risk factors for infection complications after PCNL and to provide clinicians with a decision-making basis for the early prevention and treatment of infectious complications.

## Materials and method

### Search strategy

**Fig. 1:**
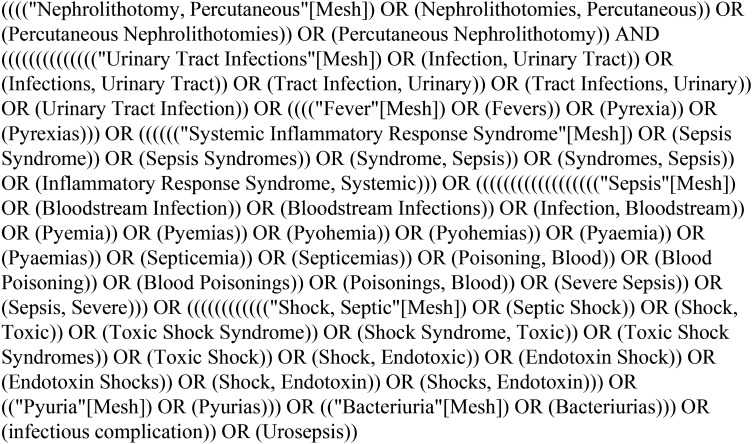
PubMed search strategy

This systematic review and meta-analysis is based on the Preferred Reporting Items for Systematic Reviews and Meta-Analyses (PRISMA) [10] for reporting. From February 1, 2022, to April 30, 2022, two researchers experienced in systematic reviews conducted a search of PubMed, Web of Science, Cochrane Library, and EMBASE to identify studies of patients receiving PCNL for upper urinary tract stones. The search strategy was initially developed in PubMed, the keywords “PCNL” and “UTI, fever, SIRS, sepsis, septic shock, bacteriuria” were searched, and the specific search strategy is shown in Fig. 1, subsequently applied to other database searches.

### Inclusion and exclusion criteria

During the screening process, we included the following studies: (I) observational studies on risk factors for postoperative infectious complications of PCNL; (II) literature with risk factors that could extract odds ratio (OR), mean difference (MD), 95% confidence interval (CI) or OR value, MD value, 95% CI could be calculated from the original data. The relevant studies were also excluded according to the exclusion criteria established in advance, which were as follows: (I) studies in which PCNL included both ureteroscopic or extracorporeal shock wave lithotripsy patients; (II) animal studies, case reports, conference proceedings, guidelines, letters, and review articles; (III) literature with incomplete or unclear data or significant errors; and (IV) literature with duplicate publications and inaccessible full text. No language or publication date restrictions were used throughout.

### Data extraction and quality assessment

Two investigators independently screened the titles, abstracts, and full texts based on the inclusion and exclusion criteria. The final list of studies used for full-text review was determined after documented comparisons and discussions. Any disagreements were resolved through consultative discussions and, if needed, with third-party investigators. These studies included at least one infectious complication of urinary tract infection (UTI), SIRS, and sepsis after PCNL. UTI was defined as the presence of a body temperature ≥ 38.5 °C within 48 h after surgery and the presence of bacteriuria within one week [11]. SIRS was determined by meeting any two of the following criteria: heart rate > 90 beats/min; respiratory rate > 20 breaths/min or PaCO2 < 32 mmHg; body temperature < 36 °C or > 38 °C; white blood cell count > 12,000/mm^3^ or < 4000/mm^3^ [12]. Sepsis was defined as a qSOFA (rapid sepsis-associated organ failure assessment) score ≥ 2 of the following scoring criteria: respiratory rate ≥ 22/min; altered mental status (Glasgow Coma Score < 13); and systolic blood pressure ≤ 100 mmHg [13].

We extracted the study period, study type, sample size, incidence of postoperative infectious complications, mean age, perioperative characteristics, and clinical characteristics of participants in the article’s postoperative noninfectious complications group and infectious complications group. In this review, we consulted relevant literature and screened all risk factors in the included studies. The final identified factors were five demographic factors, including gender, diabetes, body mass index (BMI), age, and hypertension; nine laboratory parameters, including preoperative urine culture (UC), renal pelvis urine culture (RPUC), stone culture (SC), urine leukocytes, infected stones, peripheral blood leukocytes, neutrophil-to-lymphocyte ratio (NLR), platelet-to-lymphocyte ratio (PLR), and blood creatinine; and eight perioperative factors, including staghorn stones, preoperative stenting, blood transfusion, number of channels, operation time, postoperative residual stones, stone size, and hydronephrosis. In addition, we used the Newcastle‒Ottawa scale (NOS) [14] for the quality assessment of the included studies.

### Statistical analyses

All statistical analyses were performed by RevMan (version 5.3) software. Effect sizes were calculated as the OR (95% CI) for each risk factor leading to complications of infection. If the OR was not provided in the study, the effect size was calculated based on the MD (95% CI). Heterogeneity between studies was analyzed using the Q test, and the magnitude of heterogeneity was assessed using the *I*^2^ statistic. *I*^2^ < 25%, *I*^2^ = 25–50%, and *I*^2^ > 50% represent low, moderate, and high inconsistency, respectively. At the final assessment in this study, *I*^2^ < 50% and *P* > 0.05 indicated that there was no statistical heterogeneity across studies, and a fixed-effects model was used; conversely, a random-effects model was used, and sensitivity analysis was performed to analyze the heterogeneity of ≥ 2 included studies by excluding the literature one by one for each factor. Subgroup analysis was performed according to the type of study infection complications, and effect values were estimated separately for different groups. Finally, article publication bias was assessed. *P*-values ≤ 0.05 were considered statistically significant.

## Results

**Fig. 2:**
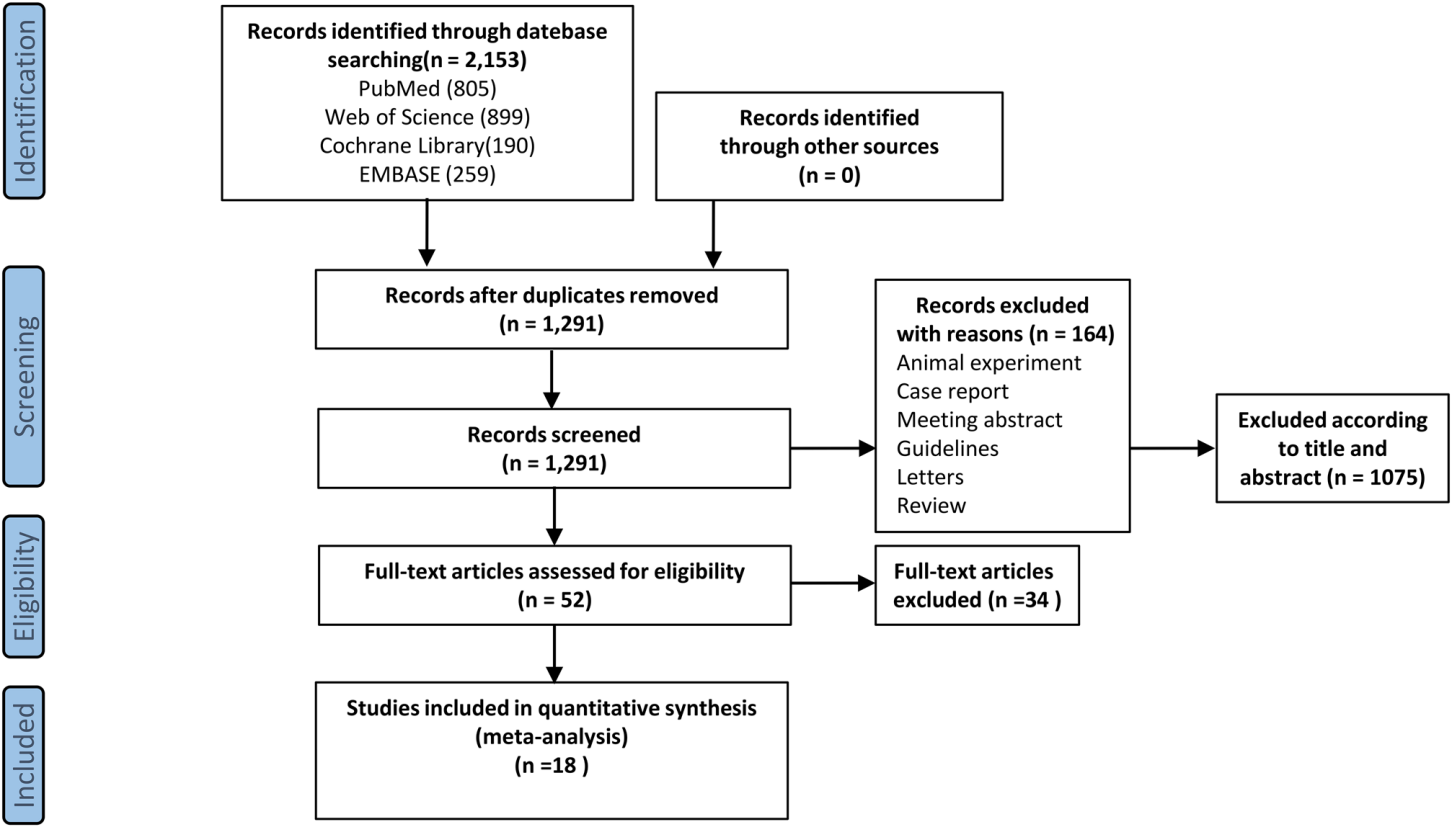
Flow diagram of study selection

Of the 2152 articles searched, 18 met the eligibility criteria and were included in the systematic evaluation [11, 15–31]. Figure 2 shows the PRISMA flowchart for study identification and selection of outcomes. Of these 18 studies conducted between 2008 and 2021, the total number of patients studied was 7161, the mean age ranged from 46.4 to 55.5 years, and the incidence of post-PCNL infectious complications ranged from 2.4% to 40.4%, with inconsistent risk factors reported across studies (Table 1). There was some variation in perioperative characteristics across studies (Table 2). Table 3 shows the results of the meta-analysis of the 22 risk factors. The results of the quality evaluation of the 18 studies by the NOS scale are shown in Table 4.

**Table 1:** Summary of the included studies

Study	Study period	Design	Sample size, *n*	Patients with infectious complications, *n* (%)	Sample overall age	Risk factors
Akdeniz et al. [15]	2015–2020	Retrospective Case–control	228	29 (12.7)	48.5	ABCQRT
Budak et al. [11]	2013–2018	Retrospective Case–control	222	38 (17.1)	52.9	ABFRT
Cetinkaya et al. [16]	2013–2015	Retrospective Case–control	192	41 (21.6)	47.3	ABCDLMRSTV
Chan et al. [17]	2005–2009	Retrospective Case–control	99	40 (40.4)	52.6	ABCDEFKNOPSTV
Chen et al. [18]	2016–2017	Retrospective Case–control	802	19 (2.4)	52.0	ABDEFJNRSV
Chen et al. [19]	2005–2007	Retrospective Case–control	209	49 (23.4)	49.1	FOQR
Erdil et al. [20]	2008–2011	Retrospective Case–control	317	53 (16.7)	47.5	ADFGHNQRSTV
Gao et al. [21]	2011–2018	Retrospective cohort	197	34 (35.1)	48.7	ABCDFKLOQRST
He et al. [22]	2014–2016	Retrospective Case–control	1030	108 (10.49)	52.1	ABCDFINOS
Koras et al. [23]		Retrospective Case–control	303	83 (27.4)	46.4	CFGHJQRSTV
Lojanapiwat et al. [24]		Retrospective Case–control	200	56 (28)	51.5	ACDFGHSTU
Peng et al. [25]	2016–2020	Retrospective Case–control	365	108 (29.6)	53.9	ABCDEFLMNSU
Sen et al. [26]		Retrospective Case–control	487	91 (18.7)	48.5	AFGHQRT
Tang et al. [27]	2016–2020	Retrospective Case–control	758	97 (12.8)	52.0	ABCDFKLNPRS
Wang et al. [28]	2017–2019	Retrospective Case–control	246	15 (6.1)	51.2	ABDFO
Xu et al. [29]	2015–2018	Retrospective Case–control	556	123 (22.1)	52.0	ABEF
Yang et al. [30]	2010–2014	Retrospective Case–control	164	45 (27.4)	51.1	ABDNOSUV
Zhu et al. [31]	2017–2019	Retrospective Case–control	786	23 (2.9)	55.5	ABDINPQRSV

A Gender, B Diabetes, C BMI, D Age, E Hypertension, F UC, G RPUC, H SC, I Urine leukocyte, J Infected stone, K Peripheral blood leukocyte, L NLR, M PLR, N Creatinine, O Staghorn stone, P Preoperative stent placement, Q Blood transfusion, R Access number, S Operative time, T Residual stone, U Stone size, V Hydronephrosis

*BMI* body mass index, *UC* urine culture, *RPUC* renal pelvis urine culture, *SC* stone culture, *NLR* neutrophil-to-lymphocyte ratio, *PLR* platelet-to-lymphocyte ratio

**Table 2:** Perioperative details among included studies

Study	Preoperative antibiotic	PCNL type	Operative time	Patients with infectious complications, *n* (%)
Akdeniz et al. [15]	( −) urine: a single dose of antibiotic prophylaxis	Standard-PCNL	60	29 (12.7)
Budak et al. [11]	( +) urine:2 weeks targeted treatment ( −) urine: Intraoperative IV cefuroxime or ciprofloxacin	Standard-PCNL	88	38 (17.1)
Cetinkaya et al. [16]	( +) urine: treated with antimicrobials	Standard-PCNL	52	41 (21.6)
Chan et al. [17]	( +) urine: treated with antimicrobials	Standard-PCNL	225.7	40 (40.4)
Chen et al. [18]	( +) urine:3–14 days targeted treatment ( −) urine: a single dose of antibiotic prophylaxis	Standard-PCNL	98.4	19 (2.4)
Chen et al. [19]	All patients:7 days ciprofloxacin 250 mg twice daily 、Intraoperative IV ceftriaxone 2.0 g	Standard-PCNL	105	49 (23.4)
Erdil et al. [20]	( +) urine:7 days targeted treatment ( −) urine: Intraoperative IV ceftriaxone 1 g	Standard-PCNL	81.7	53 (16.7)
Gao et al. [21]	( +) urine: targeted treatment ( −) urine: Preoperative IV second-generation cephalosporin	Standard-PCNL	104.1	34 (35.1)
He et al. [22]	( +) urine:7 days targeted treatment ( −) urine: Preoperative IV cefathiamidine		153	108 (10.49)
Koras et al. [23]	( +) urine:7 days targeted treatment ( −) urine: a single dose of antibiotic prophylaxis	Standard-PCNL	119.2	83 (27.4)
Lojanapiwat et al. [24]	( +) urine:2 days targeted treatment ( −) urine: Intraoperative IV ceftriaxone 2 g	Standard-PCNL	51.7	56 (28)
Peng et al. [25]	( +) urine:7 days targeted treatment ( −) urine: Preoperative IV cefamezin 1 g	Standard-PCNL	83.8	108 (29.6)
Sen et al. [26]	( +) urine:7 days targeted treatment ( −) urine: a single dose of antibiotic prophylaxis	Standard-PCNL	108.1	91 (18.7)
Tang et al. [27]	All patients: Intraoperative treated with antimicrobials	Standard-PCNL	119.6	97 (12.8)
Wang et al. [28]	( +) urine: targeted treatment	Standard-PCNL	87.9	15 (6.1)
Xu et al. [29]	( +) urine:7 days targeted treatment ( −) urine: Preoperative IV moxifloxacin 0.4 g			123 (22.1)
Yang et al. [30]	( +) urine: targeted treatment		133.7	45 (27.4)
Zhu et al. [31]	( −) urine: a single dose of antibiotic prophylaxis	Mini-PCNL	90.3	23 (2.9)

**Table 3:** Meta-analysis results of 22 risk factors

	Risk factor	No. of studies	Statistic	OR\MD (95% CI)	*P*
Demographic Factors	Gender	16	OR	1.60 [1.23, 2.07]	< 0.001
	Diabetes	13	OR	1.55 [1.10, 2.20]	= 0.01
	BMI	10	MD	− 0.56 [− 0.88, − 0.24]	< 0.001
	Age	13	MD	0.43 [− 0.62, 1.47]	= 0.42
	Hypertension	4	OR	1.15 [0.86, 1.55]	= 0.34
Laboratory Testing	UC	14	OR	3.16 [2.11, 4.74]	< 0.001
Factors	RPUC	4	OR	5.81 [1.75, 19.32]	= 0.004
	SC	4	OR	5.11 [1.46, 17.89]	= 0.01
	Urine leukocyte	2	OR	3.61 [2.45, 5.34]	< 0.001
	Infected stone	2	OR	7.00 [1.27, 38.55]	= 0.03
	Peripheral blood leukocyte	3	MD	0.71 [0.31, 1.10]	< 0.001
	NLR	4	MD	0.55 [0.43, 0.66]	< 0.001
	PLR	2	MD	19.32 [− 7.22, 45.86]	= 0.15
	Creatinine	8	MD	0.13 [− 4.72, 4.98]	= 0.96
Perioperative	Staghorn stone	6	OR	3.07 [1.32, 7.11]	= 0.009
Factors	Preoperative stent placement	3	OR	2.04 [1.07, 3.88]	= 0.03
	Blood transfusion	7	OR	3.63 [1.98, 6.65]	< 0.001
	Access number	11	OR	2.58 [1.75, 3.82]	< 0.001
	Operative time	12	MD	10.20 [4.80, 15.60]	< 0.001
	Residual stone	9	OR	1.56 [1.24, 1.98]	< 0.001
	Stone size	3	MD	0.22 [− 0.04, 0.48]	= 0.1
	Hydronephrosis	7	OR	1.21 [0.90, 1.63]	= 0.21

*OR* odds ratio, *MD* mean difference; *CI* confidence interval

**Table 4:** Newcastle–Ottawa scale literature quality evaluation

Study	Selection	Comparability	Exposure	NOS
Akdeniz et al. [15]	☆☆☆	☆	☆☆	6
Budak et al. [11]	☆☆☆	☆☆	☆☆	7
Cetinkaya et al. [16]	☆☆☆	☆	☆☆	6
Chan et al. [17]	☆☆☆		☆☆	5
Chen et al. [14]	☆☆☆	☆☆	☆☆	7
Chen et al. [15]	☆☆☆	☆☆	☆☆	7
Erdil et al. [20]	☆☆☆	☆☆	☆☆	7
Gao et al. [21]	☆☆☆	☆☆	☆☆	7
He et al. [18]	☆☆☆	☆	☆☆	6
Koras et al. [23]	☆☆	☆☆	☆☆	6
Lojanapiwat et al. [24]	☆☆	☆☆	☆☆	6
Peng et al. [25]	☆☆☆	☆	☆☆	6
Sen et al. [26]	☆☆	☆	☆☆	5
Tang et al. [27]	☆☆☆	☆	☆☆	6
Wang et al. [28]	☆☆☆	☆	☆☆	6
Xu et al. [29]	☆☆☆	☆	☆☆	6
Yang et al. [30]	☆☆☆	☆	☆☆	6
Zhu et al. [31]	☆☆☆	☆	☆☆	6

### Demographic factors

**Fig. 3:**
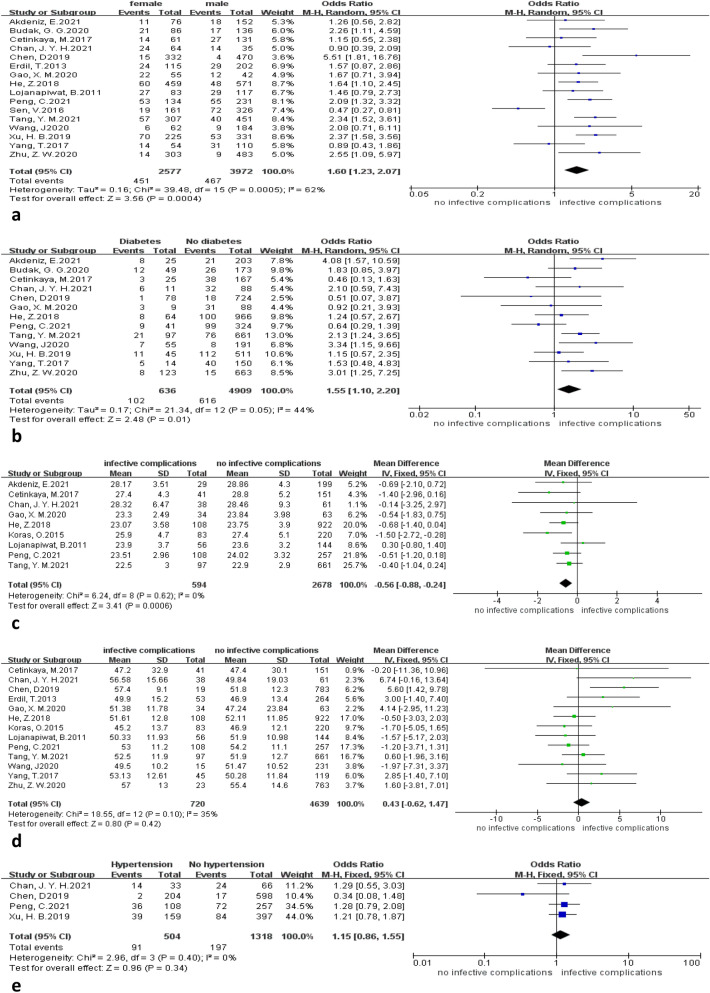
Forest plot of demographic factors. **(a)** Infectious complications in female and male. **(b)** Infectious complications with and without diabetes. **(c)** BMI difference in patients with and without infectious complication. **(d)** Age difference in patients with and without infectious complication. **(e)** Infectious complications with and without hypertension

A forest plot is provided to describe the relationship between the five demographic factors included in this study and infection complications (Fig. 3). Being female (OR = 1.60, 95% CI 1.23–2.07, *P* < 0.001) and having a history of diabetes (OR = 1.55, 95% CI 1.10–2.20, *P* = 0.01) were identified as risk factors for the development of infectious complications after PCNL. Patients with infectious complications had a lower BMI than those without infectious complications (MD = − 0.56, 95% CI − 0.88 to − 0.24, *P* < 0.001).

Age (MD = 0.43, 95% CI − 0.62–1.47, *P* = 0.42) and hypertension (OR = 1.15, 95% CI 0.86–1.55, *P* = 0.34) were not found to be associated with infectious complications.

### Laboratory testing factors

**Fig. 4:**
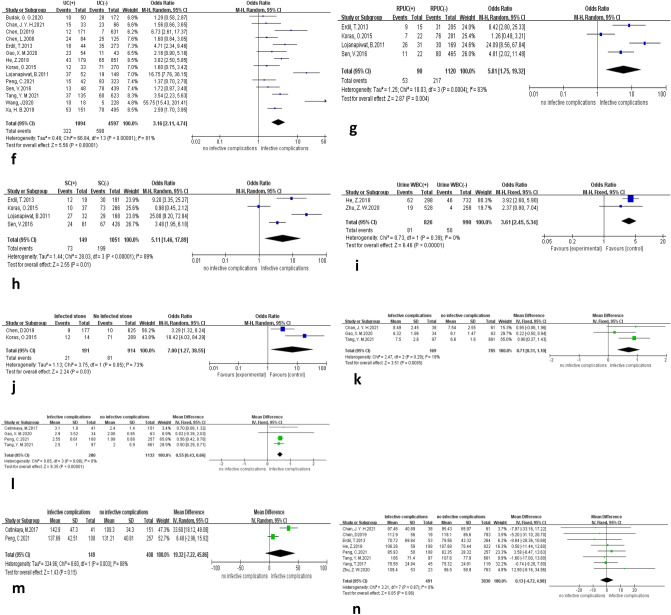
Forest plot of laboratory testing factors. **(f)** Infectious complications of positive and negative urine cultures. **(g)** Infectious complications of positive and negative renal pelvis urine culture. **(h)** Infectious complications of positive and negative stone culture. **(i)** Infectious complications of positive and negative urine leukocyte. **(j)** Infectious complications with and without infected stones. **(k)** Blood leukocyte difference in patients with and without infectious complication. **(l)** Neutrophil-to-lymphocyte ratio difference in patients with and without infectious complication. **(m)** Platelet-to-lymphocyte ratio difference in patients with and without infectious complication. **(n)** Creatinine difference in patients with and without infectious complication

A forest plot of the relationship between nine laboratory test factors and post-PCNL infection complications is shown in Fig. 4. By meta-analysis positive preoperative UC (OR = 3.16, 95% CI 2.11–4.74, *P* < 0.001), positive RPUC (OR = 5.81, 95% CI 1.75–19.32, *P* = 0.004), positive stone culture (OR = 5.11, 95% CI 1.46–17.89, *P* = 0.01), urine leukocyte positivity (OR = 3.61, 95% CI 2.45–5.34, *P* < 0.001), and infected stones (OR = 7.00, 95% CI 1.27–38.55, *P* = 0.03) were risk factors for infectious complications after PCNL, where sensitivity analysis of UC, RPUC, and SC did not change the results of meta-analysis (Table 5). Patients with infectious complications had higher blood leukocyte counts (MD = 0.71, 95% CI 0.31–1.10, *P* < 0.001) and NLR (MD = 0.55, 95% CI 0.43–0.66, *P* < 0.001) than patients with noninfectious complications.

**Table 5:** Heterogeneity and sensitivity analysis of risk factors associated with infectious complications

	Research factors	Remove documents	Before removing					After removing				
			I^2^	*P*	Model	OR\MD (95% CI)	*P*	I^2^	*P*	Model	OR\MD (95% CI)	*P*
Demographic	Gender	Sen et al. [26]	62%	＜0.001	Random Effect Model	1.60 [1.23, 2.07]	< 0.001	21%	= 0.22	Random Effect Model	1.79 [1.49, 2.15]	< 0.001
Factors	Diabetes	Peng et al. [25]	44%	= 0.05	Random Effect Model	1.55[1.10, 2.20]	= 0.01	28%	= 0.17	Random Effect Model	1.73 [1.25, 2.39]	< 0.001
Laboratory Testing	UC	None	81%	＜0.001	Random Effect Model	3.16 [2.11, 4.74]	< 0.001	81%	＜0.001	Random Effect Model	3.16 [2.11, 4.74]	< 0.001
Factors	RPUC	Koras et al. [23] and Lojanapiwat et al. [24]	83%	＜0.001	Random Effect Model	5.81 [1.75, 19.32]	=0.004	0%	=0.43	Random Effect Model	5.97 [3.01, 11.81]	< 0.001
	SC	Koras et al. [23] and Sen et al. [26]	89%	＜0.001	Random Effect Model	5.11 [1.46, 17.89]	=0.01	50%	=0.16	Random Effect Model	15.34 [5.53, 42.56]	＜0.001
Perioperative	Staghorn stone	Wang et al. [28]	83%	＜0.001	Random Effect Model	3.07 [1.32, 7.11]	=0.009	27%	=0.24	Random Effect Model	2.00 [1.35, 2.97]	< 0.001
Factors	Blood transfusion	Chen et al. [19]	52%	=0.05	Random Effect Model	3.63 [1.98, 6.65]	< 0.001	19%	=0.29	Random Effect Model	2.92 [1.85, 4.61]	< 0.001
	Access number	Cetinkaya et al. [16] and Chen et al. [19]and Erdil et al. [20]	59%	=0.006	Random Effect Model	2.58 [1.75, 3.82]	< 0.001	0%	=0.57	Random Effect Model	2.34 [1.79, 3.05]	< 0.001
	Operative time	Koras et al. [23] and Lojanapiwat [24]	64%	=0.001	Random Effect Model	10.20 [4.80, 15.60]	< 0.001	43%	=0.07	Random Effect Model	10.08 [4.98, 15.18]	< 0.001
	Stone size	Peng et al. [25]	51%	=0.31	Random Effect Model	0.22 [-0.04, 0.48]	=0.1	21%	=0.57	Random Effect Model	0.38 [0.03, 0.73]	=0.03

There was no statistically significant difference in PLR (MD = 19.32, 95% CI − 7.22–45.86, *P* = 0.15) or creatinine (MD = 0.13, 95% CI − 4.72–4.98, *P* = 0.96) in patients with infectious complications compared to patients without infectious complications.

### Perioperative factors

**Fig. 5:**
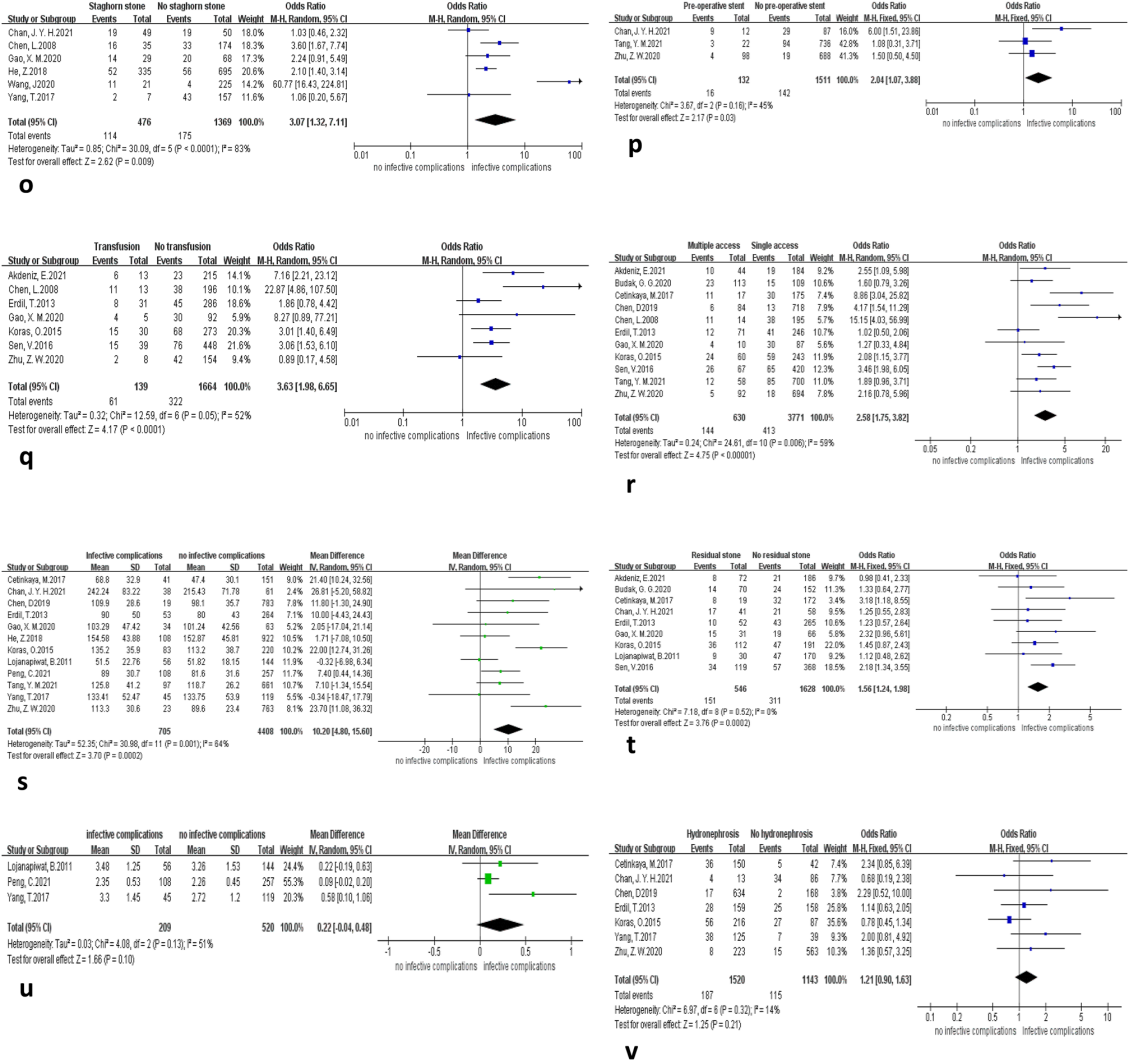
Forest plot of perioperative factors. **(o)** Infectious complications of with and without staghorn stone. **(p)** Infectious complications of with and without preoperative stent placement. **(q)** Infectious complications of with and without blood transfusion. **(r)** Infectious complications of multiple and single access number. **(s)** Operative time difference in patients with and without infectious complication. **(t)** Infectious complications of with and without residual stone. **(u)** Stone size difference in patients with and without infectious complication. **(v)** Infectious complications of with and without hydronephrosis

The forest plot of postoperative infectious complications after PCNL with eight perioperative influencing factors is shown in (Fig. 5). Among them, staghorn stones (OR = 3.07, 95% CI 1.32–7.11, *P* = 0.009), preoperative stenting (OR = 1.55, 95% CI 1.10–2.20, *P* = 0.01), blood transfusion (OR = 3.63, 95% CI 1.98–6.65, *P* < 0.001), multiple puncture access (OR = 2.58, 95% CI 1.75–3.82, *P* < 0.001), and postoperative residual stones (OR = 1.56, 95% CI 1.24–1.98, *P* < 0.001) were identified as risk factors for the development of infectious complications. Patients with infectious complications had longer operative times than those without infectious complications (MD = 10.20, 95% CI 4.80–15.60, *P* < 0.001).

The meta-analysis showed no statistically significant difference in stone size in patients with infectious complications compared to patients without infectious complications (MD = 0.22, 95% CI − 0.04–0.48, *P* = 0.1), with high heterogeneity (*I*^2^ = 51%). In the sensitivity analysis, heterogeneity disappeared, and the meta-analysis results changed when the Peng study was removed (MD = 0.38, 95% CI 0.03–0.73, *P* = 0.03), and stone size may be associated with the development of infectious complications.

The association between hydronephrosis (OR = 1.21, 95% CI 0.90–1.63, *p* = 0.21) and infectious complications was not statistically significant.

### Subgroup analysis

A subgroup analysis was performed to classify the included postoperative infectious complications (Table 6). The results showed no statistically significant association between a history of diabetes mellitus and different infectious complications; there was no statistically significant association between staghorn stones, blood transfusion, and postoperative sepsis. The results of the other factor subgroup analysis did not change compared to the meta-analysis results.

**Table 6:** Subgroup analysis of risk factors

Risk factor	Subgroup	No. of studies, *n*	Heterogeneity I^2^, %p value	OR/MD (95% CI)	*p* value
Gender	Urosepsis	4	00.4	2.48 [1.54, 3.99]	< 0.01
	SIRS	11	69 < 0.01	1.40 [1.03, 1.89]	0.03
	FUTI	1	NANA	2.26 [1.11, 4.59]	0.02
Diabetes	Urosepsis	4	350.2	1.97 [0.91, 4.26]	0.09
	SIRS	8	530.04	1.39 [0.89, 2.17]	0.15
	FUTI	1	NANA	1.83 [0.85, 3.97]	0.12
BMI	Urosepsis	1	NANA	-0.54 [-1.83, 0.75]	0.41
	SIRS	8	00.51	-0.56 [-0.90, -0.23]	< 0.01
Age	Urosepsis	4	410.16	2.65 [0.04, 5.27]	0.05
	SIRS	9	210.26	0.00 [-1.14, 1.14]	0.99
Hypertension	Urosepsis	1	NANA	0.34 [0.08, 1.48]	0.15
	SIRS	3	00.98	1.25 [0.92, 1.69]	0.15
UC	Urosepsis	3	88 < 0.01	8.82 [1.56, 49.94]	0.01
	SIRS	10	77 < 0.01	2.78 [1.87, 4.14]	< 0.01
	FUTI	1	NANA	1.29 [0.58, 2.87]	0.54
RPUC	SIRS	4	83 < 0.01	5.81 [1.75, 19.32]	< 0.01
SC	SIRS	4	89 < 0.01	5.11 [1.46, 17.89]	0.01
Urine leukocyte	Urosepsis	1	NANA	2.37 [0.80, 7.04]	0.12
	SIRS	1	NANA	3.92 [2.60, 5.90]	< 0.01
Infected stone	Urosepsis	1	NANA	3.29 [1.32, 8.24]	0.01
	SIRS	1	NANA	18.42 [4.03, 84.29]	< 0.01
Peripheral blood leukocyte	Urosepsis	1	NANA	0.22 [-0.50, 0.94]	0.55
	SIRS	2	00.93	3.29 [1.32, 8.24]	< 0.01
NLR	Urosepsis	1	NANA	0.82 [-0.39, 2.03]	0.18
	SIRS	3	00.8	0.22 [-0.50, 0.94]	< 0.01
PLR	SIRS	2	88 < 0.01	19.32 [-7.22, 45.86]	0.15
Creatinine	Urosepsis	2	80.3	5.29 [-11.50, 22.08]	0.54
	SIRS	6	00.89	-0.34 [-5.40, 4.73]	0.9
Staghorn stone	Urosepsis	2	94 < 0.01	11.27 [0.43, 295.33]	0.15
	SIRS	4	450.14	5.29 [-11.50, 22.08]	0.01
Preoperative stent placement	Urosepsis	1	NANA	1.50 [0.50, 4.50]	0.47
	SIRS	2	700.07	2.42 [1.08, 5.42]	0.03
Blood transfusion	Urosepsis	2	600.11	2.37 [0.27, 20.91]	0.44
	SIRS	5	580.05	3.99 [2.09, 7.61]	< 0.01
Access number	Urosepsis	3	60.34	2.48 [1.30, 4.76]	< 0.01
	SIRS	7	72 < 0.01	2.93 [1.70, 5.03]	< 0.01
	FUTI	1	NANA	1.60 [0.79, 3.26]	0.19
Operative time	Urosepsis	3	480.15	9.19 [3.13, 15.25]	0.02
	SIRS	9	67 < 0.01	9.19 [3.13, 15.25]	< 0.01
Residual stone	Urosepsis	1	NANA	2.32 [0.96, 5.61]	0.06
	SIRS	7	40.4	1.54 [1.19, 2.00]	< 0.01
	FUTI	1	NANA	1.33 [0.64, 2.77]	0.44
Stone size	SIRS	3	510.13	0.22 [-0.04, 0.48]	0.1
Hydronephrosis	Urosepsis	2	00.55	1.61 [0.77, 3.36]	0.2
	SIRS	5	330.2	1.14 [0.83, 1.58]	0.42

NA = not available

### Publication bias

**Fig. 6:**
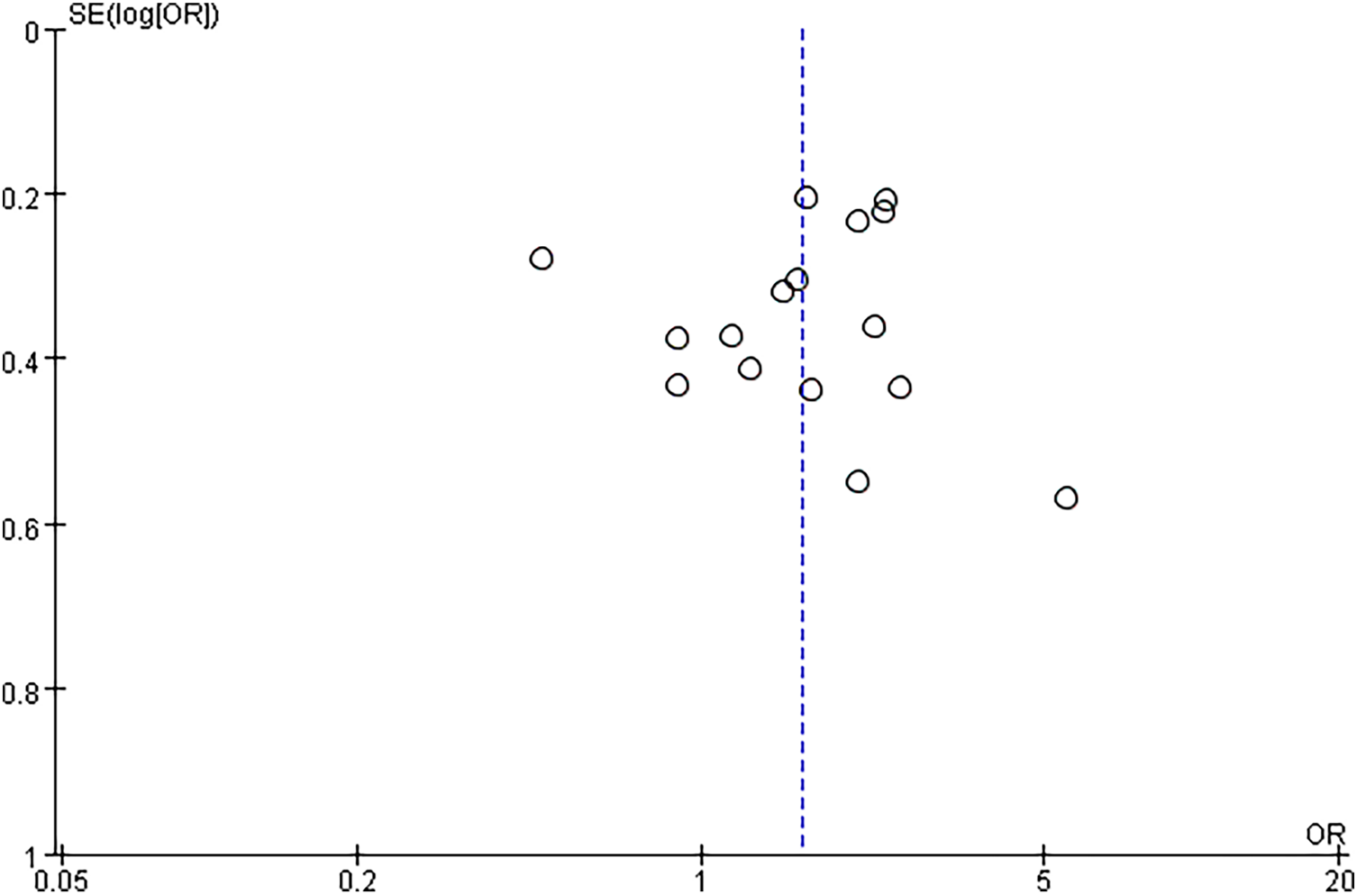
Bias analysis results of risk factors related to infectious complications

The publication bias of the articles was analyzed using gender as a proxy for risk factors. As shown in Fig. 6, the literature data were evenly distributed on both sides of the null line, indicating that the publication bias was insignificant and the included data were robust. The included data were trustworthy and reliable.

## Discussion

PCNL is gradually becoming the primary method of treating large upper urinary tract stones. Although PCNL is a minimally invasive procedure, there is a risk of infectious complications such as systemic inflammatory response syndrome and sepsis, which can be life-threatening in severe cases. Therefore, early identification of risk factors for infectious complications is a crucial measure to prevent these complications. Many studies have shown that gender, age, hypertension, diabetes, positive UC, stone size, deerstalker-shaped stones, and time of surgery are factors associated with the development of infectious complications after PCNL; however, there is controversy among these studies. Therefore, this review analyzed the 22 most common risk factors in the past, including five demographic factors, nine laboratory test factors, and eight perioperative factors.

The corresponding results were obtained after META-analysis (Table 3), sensitivity analysis (Table 5), and subgroup analysis (Table 6). Here, we will discuss the factors that had a meaningful impact on the analysis in three aspects: demographic factors, laboratory test factors, and perioperative factors.

### Demographic factors

In this study, we found that the incidence of infectious complications after PCNL in women was higher (Fig. 3a), probably because women have a shorter urethra and the urethral opening is close to the vagina and anus, which are more susceptible to infection. Second, due to the decrease in estrogen levels in climacteric women, the urinary tract mucosa will atrophy, resulting in a decrease in glycogen in epithelial cells, a corresponding decrease in glycogen-dependent vaginal flora, and a corresponding increase in Escherichia coli, leading to urinary tract infection [32–34]. Previous studies have shown that women are more prone to infectious complications after PCNL [3]. Based on our findings and previous studies, we can conclude that women are an independent risk factor for post-PCNL infections.

Studies have shown that the high blood sugar status of diabetic patients lends itself to bacterial growth, leading to frequent episodes of urinary tract infections [35]. Second, chronic hyperglycemia also decreases the mobility, chemotaxis, phagocytosis, and adhesion of leukocytes, monocytes, and macrophages, thus reducing the immunity and resistance of the body [36]. A study by Jia et al. showed that diabetic patients had increased expression of peripheral blood T-cell programmed death factor 1 (PD-1), inhibiting T-cell function and proliferation and suppressing cellular immunity [37]. This meta-analysis concludes that a history of diabetes increases the incidence of postoperative infectious complications in PCNL (Fig. 3b). However, the association between a history of diabetes and different infectious complications in the subgroup analysis (Table 6) was not statistically significant, and further studies are needed to draw valid conclusions. Therefore, this study can only cautiously conclude that a history of diabetes may be a risk factor for post-PCNL infectious complications.

The present meta-analysis showed that patients with infectious complications had a lower BMI than those without infectious complications (Fig. 3c). Alhabeeb et al. [38] in a recent study of the relationship between BMI and urinary tract infections concluded that obese individuals had a significantly increased risk of urinary tract infections compared to those of normal weight, while overweight individuals had no significant increase in the risk of urinary tract infections; underweight individuals had no significant decrease in the risk of urinary tract infections. Therefore, based on our findings and previous studies, we hypothesize that among those with a BMI less than 25, those with a smaller BMI may have poorer immune function than those with a larger BMI; therefore, those with a low BMI after PCNL are more likely to develop urinary tract infections.

### Laboratory testing factors

Our review concluded that UC, RPUC, and SC positivity are risk factors for infectious complications (Figs. 3h, 4f). The failure to reduce UC heterogeneity after sensitivity analysis by excluding the literature one by one, we considered the variation due to the different microbiological testing methods and time of testing used by different healthcare institutions in the included studies. First, the predictive value of UC for postoperative infection in PCNL in previous studies is controversial because various infectious complications may occur after PCNL despite negative culture results or prophylactic antibiotic treatment based on UC. Some studies have suggested that a positive UC is a critical factor for SIRS and sepsis after PCNL [39]. In contrast, in the study by Walton-Diaz et al., no postoperative infection occurred in UC-positive patients, and UC-negative does not accurately reflect the microbiological status of the upper urinary tract. Therefore, UC does not seem to be a good predictor of infection after PCNL [40]. Second, many studies have reported the importance of RPUC and SC [3, 40–42]. Although intraoperative RPUC and SC results are sometimes inconsistent, they may be the only way to identify infecting microorganisms to adjust antimicrobial therapy. Positive intraoperative cultures are more likely to have postoperative infectious complications. In this study, we concluded that positive UC, RPUC, and SC increased the incidence of postoperative infectious complications in PCNL. We believe that although it is more accurate to use RUBC and SC to predict infection after PCNL, the value of early prediction is greatly limited because of the lengthy culture time. Therefore, although the false-negative rate of UC is high, as long as preoperative UC is positive, we can better predict the occurrence of infection. Preoperative UC is still a better predictor that can be obtained early.

This study shows that positive urine leukocytes increase the incidence of infectious complications (Fig. 4i). Several previous studies have explored the relationship between urine leukocytes and infectious complications. First, Chen et al. [18] One study reported a higher rate of postoperative infections in patients with WBC ≥ 2 + than in patients with WBC < 2 + . Second, Ruan et al.’s [43] meta-analysis showed that preoperative positive urinary leukocytes were an independent risk factor for postoperative infection in PCNL. This is consistent with our results that urine leukocyte positivity is a risk factor for postoperative infectious complications in PCNL.

Infectious stones account for approximately 10% of all urinary calculi 15% [44]. Studies have shown that infectious stones are produced in the presence of urea-lytic bacteria, and the higher its content is, the higher the frequency of urea-lytic bacteria in urine culture and the higher the incidence of postoperative infectious complications in patients [44, 45]. In addition, infected stones contain higher levels of endotoxin than noninfected stones [46]. During the crushing of PCNL stones, colonized bacteria and bacterial endotoxins are released from the stones. Bacteria and endotoxins may enter the body circulation due to hydrostatic pressure generated by renal perfusion fluid and are more likely to develop systemic inflammatory responses. According to the results of this study (Fig. 4j), we can conclude that infectious stones are risk factors for infection after PCNL.

Our review found that patients with infectious complications had higher blood leukocytes than those with noninfectious complications (Fig. 4k). We consider this because leukocytes are cells with defensive functions. When a foreign infection occurs, granulocytes in the bone marrow are released into the peripheral blood, causing an increase in leukocytes in the peripheral blood to actively participate in the defensive response against the infection. Therefore, leukocytes increase during bacterial infections, and the more severe the bacterial infection is, the more pronounced the leukocyte increase. We are not aware of any previous studies that have systematically addressed the relationship between peripheral blood leukocytes and postoperative infection complications in PCNL. The results of this review may provide a new theoretical basis for the early prevention of postoperative infectious complications in PCNL.

Some scholars have studied the potential molecular basis of NLR associated with infectious complications and found that plasma levels of proinflammatory cytokines are increased in patients with elevated NLR. These inflammatory cytokines accumulate in the tissue microenvironment and can lead to invasive inflammation [47]. Based on the present review, we conclude that patients with infectious complications have a higher NLR than patients without infectious complications (Fig. 4l). We believe that NLR is expected to be an early predictor of postoperative infection in PCNL due to the ease and speed of detection.

### Perioperative factors

The present meta-analysis showed that staghorn stones increase the risk of post-PCNL infectious complications (Fig. 5o). We consider that staghorn stones have a large stone load, leading to a prolonged surgical lithotripsy time, which indirectly leads to an increased rate of infectious complications. Most staghorn stones contain magnesium ammonium phosphate, calcium carbonate, and apatite [22], which are infectious stones. In the previous discussion, we concluded that infectious stones significantly increase the risk of postoperative infectious complications. Rivera et al. also showed that antler-shaped stones increased the risk of postoperative infection-related complications more than threefold [8]. In the subgroup analysis of the current study, the association between staghorn stones and postoperative sepsis was not statistically significant (Table 6), and further studies are still needed to draw valid conclusions. Caution is needed to conclude from the present study that staghorn stones may be a risk factor for postoperative infection in PCNL.

Our study concluded that preoperative stenting increases the risk of postoperative infectious complications in PCNL (Fig. 5p). We believe that after insertion of the stent, some urine components form regulatory membranes along the surface of the tube, a phenomenon that alters the characteristics of the catheter surface and provides the necessary conditions for the adhesion of pathogenic microorganisms. Ureteral stents are tubes that connect the bladder to the renal pelvis, and microorganisms move against the urine stream during operation, rise from the bladder to the kidney, and may even reach the blood [48]. Based on our results and previous studies, it is possible to conclude that preoperative ureteral stenting is a risk factor for developing postoperative infectious complications in PCNL.

The present meta-analysis concludes that blood transfusion increases the risk of postoperative infectious complications in PCNL (Fig. 5q). Allogeneic blood transfusions have repeatedly been shown to be associated with poor patient prognosis, including an increased risk of various infections in transfusion recipients. The underlying mechanisms may be related to the immunosuppressive effects of allogeneic blood iron content and bacterial contamination [49]. Sihler’s study [50] reported a twofold to sixfold increase in SIRS in trauma patients who received blood transfusions compared to those who did not. Interestingly, according to our subgroup analysis, blood transfusion increased the risk of developing postoperative SIRS. However, the relationship between transfusion and postoperative sepsis in PCNL was not statistically significant (Table 6), considering that the inclusion of fewer sepsis articles caused it. Therefore, we can only cautiously conclude that blood transfusion may be a risk factor for infectious complications.

The predictive value of intraoperative multiple puncture channels for postoperative PCNL infection is currently controversial, with previous Korets and Aron et al. [3, 51] found that multiple puncture channels were a risk factor for the development of SIRS after percutaneous nephrolithotomy. However, Erdil [20] showed that the development of post-PCNL infectious complications was not associated with multiple puncture channels. Our study showed that multiple puncture channels increase the incidence of post-PCNL infectious complications (Fig. 5r), and we conclude that more puncture channels will increase the possibility of bacterial entry into the urinary system and that multiple puncture channels are a risk factor for post-PCNL infectious complications.

This study showed that patients with infectious complications had a significantly longer operative time than patients without infectious complications (Fig. 5s). We believe that the longer operation time leads to long-term high pressure in the renal pelvis, renal pelvic venous reflux, and an increased chance of irrigating fluid absorption [19]. Zhong et al. [52] found that patients with sustained intrapelvic pressure > 30 mmHg for more than 30 s were more likely to develop postoperative infection. Based on the results of this meta-analysis, we have good reason to conclude that longer operative times are more likely to lead to postoperative infectious complications.

Our findings show that postoperative residual stones increase the risk of infectious complications (Fig. 5t). It has been found that the fragments remaining after PCNL in large and complex stones may contain bacteria and endotoxins that reach the blood vessels through the damaged endothelium, causing the dissemination of microorganisms and eventually infectious complications [6, 53]. Gutierrez et al. [7] Additionally, the presence of postoperative residual stone fragments was associated with postoperative infectious complications. However, some studies found no significant association between residual stones and infectious complications [20]. We believe that residual stone fragments may cause persistent urinary tract infections and that residual fragments from patients with positive preoperative and intraoperative cultures may act as foci of infection and contribute to the development of postoperative infectious complications. Based on our results, we conclude that postoperative residual stones are a risk factor for postoperative infectious complications of PCNL.

In our meta-analysis, there was no significant difference in stone size between patients with infectious and without infectious complications (Fig. 5u). However, in the sensitivity analysis (Table 5), the heterogeneity disappeared after Peng et al.’s study was removed, and the results of the meta-analysis changed (MD = 0.38, 95% CI 0.03–0.73, *p* = 0.03). We considered that the heterogeneity existed due to differences in the stone measurement criteria. There is a strong relationship between stone size and post-PCNL infection; when stone size increases, urinary obstruction also increases, as does the difficulty of the procedure, the duration of the procedure, and the loss of hemoglobin, and previous studies have indicated that these factors increase the risk of infectious complications [15]. Lojanapiwat and Wang [24, 28] showed that large stones resulted in a longer operative time and an increased incidence of sepsis compared to small stones. Based on the results of this study and the analysis of previous studies, we can cautiously conclude that stone size may be associated with the occurrence of infectious complications after PCNL.

### Strengths and limitations

Many studies have separately investigated the influencing factors of UTI, SIRS and sepsis after PCNL. Therefore, META-analysis of three infectious complications was performed simultaneously after unifying the diagnostic criteria in this study. Good results were obtained, and 12 independent risk factors were identified.

This study has good guiding significance for clinical work, such as preoperative UC positivity, urine leukocyte positivity, elevated peripheral blood positivity, and elevated NLR patients. We need anti-infective treatment to ensure that these indicators tend to be normal. In patients with female and preoperative stent implantation, the duration of preoperative anti-infective treatment can be relatively prolonged. Patients with RPUC positivity, SC positivity, infected stones, multiple puncture channels, prolonged operation time, and postoperative residual stones found after surgery should also be more closely detected for various infection indicators in addition to conventional anti-infective treatment to avoid the occurrence and further aggravation of postoperative infection.

Our study has some limitations. First, all studies that met our inclusion criteria were observational studies with relatively more bias and high heterogeneity in some risk factors. Second, the current review did not include more factors affecting post-PCNL infection, such as IL-6, PCT, CRP, stone load, amount of hemoglobin drop, size of the puncture channel, amount of bleeding, amount of intraoperative lavage, and renal perfusion pressure. Scholars in previous studies have discussed these factors, but due to the small number of studies, this study cannot be included in the meta-analysis. Therefore, further research is needed to establish a more accurate and effective evaluation system.

## Conclusions

Twelve factors were identified as independent risk factors for infection complications after PCNL, including female, UC positivity, RPUC positivity, SC positivity, urine leukocyte positivity, infected stones, elevated peripheral blood leukocytes, elevated NLR, preoperative stent implantation, multiple puncture channels, prolonged operation time, and postoperative residual stones. Five factors, including diabetes mellitus, BMI, staghorn stones, blood transfusion, and stone size, may be related to infection complications, and further research is needed to draw an effective conclusion. Age, hypertension, PLR, creatinine, and hydronephrosis were not associated with infectious complications.

## Data Availability

The datasets generated during and/or analyzed during the current study are available from the corresponding author on reasonable request.

## Abbreviations

PCNL: Percutaneous nephrolithotomy
OR: Odds ratio
MD: Mean difference
CI: Confidence Interval
UC: Urine culture
RPUC: Renal pelvis urine culture
SC: Stone culture
NLR: Neutrophil-to-lymphocyte ratio
BMI: Body mass index
UTI: Urinary tract infection
SIRS: Systemic inflammatory response syndrome
PRISMA: Preferred reporting items for systematic reviews and meta-analyses
PLR: Platelet-to-lymphocyte ratio
NOS: Newcastle–Ottawa scale

## References

[1] Assimos D, Krambeck A, Miller NL, Monga M, Murad MH, Nelson CP (2016). Surgical management of stones: American urological association/endourological society guideline, PART I. J Urol.

[2] de la Rosette J, Assimos D, Desai M, Gutierrez J, Lingeman J, Scarpa R (2011). The clinical research office of the endourological society percutaneous nephrolithotomy global study: indications, complications, and outcomes in 5803 patients. J Endourol.

[3] Korets R, Graversen JA, Kates M, Mues AC, Gupta M (2011). Post-percutaneous nephrolithotomy systemic inflammatory response: a prospective analysis of preoperative urine, renal pelvic urine and stone cultures. J Urol.

[4] Whitehurst L, Jones P, Somani BK (2019). Mortality from kidney stone disease (KSD) as reported in the literature over the last two decades: a systematic review. World J Urol.

[5] Yu J, Guo B, Yu J, Chen T, Han X, Niu Q (2020). Antibiotic prophylaxis in perioperative period of percutaneous nephrolithotomy: a systematic review and meta-analysis of comparative studies. World J Urol.

[6] Draga RO, Kok ET, Sorel MR, Bosch RJ, Lock TM (2009). Percutaneous nephrolithotomy: factors associated with fever after the first postoperative day and systemic inflammatory response syndrome. J Endourol.

[7] Gutierrez J, Smith A, Geavlete P, Shah H, Kural AR, de Sio M (2013). Urinary tract infections and post-operative fever in percutaneous nephrolithotomy. World J Urol.

[8] Rivera M, Viers B, Cockerill P, Agarwal D, Mehta R, Krambeck A (2016). Pre- and postoperative predictors of infection-related complications in patients undergoing percutaneous nephrolithotomy. J Endourol.

[9] Santanapipatkul K (2020). Factors associated with urosepsis following percutaneous nephrolithotomy. Int J Urol.

[10] Liberati A, Altman DG, Tetzlaff J, Mulrow C, Gøtzsche PC, Ioannidis JP (2009). The PRISMA statement for reporting systematic reviews and meta-analyses of studies that evaluate healthcare interventions: explanation and elaboration. BMJ (Clinical research ed).

[11] Budak GG, Budak S, Yucel C, Kisa E, Kozacioglu Z (2020). Risk factors for urinary tract infections after one stage percutaneous nephrolithotomy. Kuwait Medical Journal.

[12] Levy MM, Fink MP, Marshall JC, Abraham E, Angus D, Cook D (2003). 2001 SCCM/ESICM/ACCP/ATS/SIS international sepsis definitions conference. Intensive Care Med.

[13] Singer M, Deutschman CS, Seymour CW, Shankar-Hari M, Annane D, Bauer M (2016). The third international consensus definitions for sepsis and septic shock (Sepsis-3). JAMA.

[14] Stang A (2010). Critical evaluation of the Newcastle-Ottawa scale for the assessment of the quality of nonrandomized studies in meta-analyses. Eur J Epidemiol.

[15] Akdeniz E, Ozturk K, Ulu MB, Gur M, Caliskan ST, Sehmen E (2021). Risk factors for systemic inflammatory response syndrome in patients with negative preoperative urine culture after percutaneous nephrolithotomy. J Coll Phys Surg-Pak: JCPSP.

[16] Cetinkaya M, Buldu I, Kurt O, Inan R (2017). Platelet-to-lymphocyte ratio: a new factor for predicting systemic inflammatory response syndrome after percutaneous nephrolithotomy. Urol J.

[17] Chan JYH, Wong VKF, Wong JL, Paterson RF, Lange D, Chew BH (2021). Predictors of urosepsis in struvite stone patients after percutaneous nephrolithotomy. Investig Clin Urol.

[18] Chen D, Jiang CH, Liang XF, Zhong FL, Huang J, Lin YP (2019). Early and rapid prediction of postoperative infections following percutaneous nephrolithotomy in patients with complex kidney stones. BJU Int.

[19] Chen L, Xu QQ, Li JX, Xiong LL, Wang XF, Huang XB (2008). Systemic inflammatory response syndrome after percutaneous nephrolithotomy: an assessment of risk factors. Int J Urol: Offi J Jpn Urol Assoc.

[20] Erdil T, Bostanci Y, Ozden E, Atac F, Yakupoglu YK, Yilmaz AF (2013). Risk factors for systemic inflammatory response syndrome following percutaneous nephrolithotomy. Urolithiasis.

[21] Gao XM, Lu CY, Xie F, Li L, Liu M, Fang ZY (2020). Risk factors for sepsis in patients with struvite stones following percutaneous nephrolithotomy. World J Urol.

[22] He Z, Tang F, Lei H, Chen Y, Zeng G (2018). Risk factors for systemic inflammatory response syndrome after percutaneous nephrolithotomy. Progres en urologie : journal de l'Association francaise d'urologie et de la Societe francaise d'urologie.

[23] Koras O, Bozkurt IH, Yonguc T, Degirmenci T, Arslan B, Gunlusoy B (2015). Risk factors for postoperative infectious complications following percutaneous nephrolithotomy: a prospective clinical study. Urolithiasis.

[24] Lojanapiwat B, Kitirattrakarn P (2011). Role of preoperative and intraoperative factors in mediating infection complication following percutaneous nephrolithotomy. Urol Int.

[25] Peng C, Li JL, Xu G, Jin J, Chen JJ, Pan SH (2021). Significance of preoperative systemic immune-inflammation (SII) in predicting postoperative systemic inflammatory response syndrome after percutaneous nephrolithotomy. Urolithiasis.

[26] Sen V, Bozkurt IH, Aydogdu O, Yonguc T, Yarimoglu S, Sen P (2016). Significance of preoperative neutrophil-lymphocyte count ratio on predicting postoperative sepsis after percutaneous nephrolithotomy. Kaohsiung J Med Sci.

[27] Tang YM, Zhang C, Mo CQ, Gui CP, Luo JH, Wu RP (2021). Predictive model for systemic infection after percutaneous nephrolithotomy and related factors analysis. Front Surg.

[28] Wang J, Mi YY, Wu S, Shao HB, Zhu LJ, Dai F (2020). Impact factors and an efficient nomogram for predicting the occurrence of sepsis after percutaneous nephrolithotomy. BioMed Res Int.

[29] Xu HB, Hu LK, Wei XD, Niu J, Gao YY, He J (2019). The predictive value of preoperative high-sensitive C-reactive protein/albumin ratio in systemic inflammatory response syndrome after percutaneous nephrolithotomy. J Endourol.

[30] Yang T, Liu SH, Hu JM, Wang LJ, Jiang HW (2017). The evaluation of risk factors for postoperative infectious complications after percutaneous nephrolithotomy. BioMed Res Int.

[31] Zhu ZW, Cui Y, Zeng HM, Li YC, Zeng F, Li Y (2020). The evaluation of early predictive factors for urosepsis in patients with negative preoperative urine culture following mini-percutaneous nephrolithotomy. World J Urol.

[32] Della Torre S, Maggi A (2017). Sex differences: a resultant of an evolutionary pressure?. Cell Metab.

[33] Bowling MR, Xing D, Kapadia A, Chen YF, Szalai AJ, Oparil S (2014). Estrogen effects on vascular inflammation are age dependent: role of estrogen receptors. Arterioscler Thromb Vasc Biol.

[34] Jung C, Brubaker L (2019). The etiology and management of recurrent urinary tract infections in postmenopausal women. Climacteric: J Int Menopause Soc.

[35] Fu AZ, Iglay K, Qiu Y, Engel S, Shankar R, Brodovicz K (2014). Risk characterization for urinary tract infections in subjects with newly diagnosed type 2 diabetes. J Diabetes Complicat.

[36] Trevelin SC, Carlos D, Beretta M, da Silva JS, Cunha FQ (2017). Diabetes mellitus and sepsis: a challenging association. Shock (Augusta, Ga).

[37] Jia Y, Zhao Y, Li C, Shao R (2016). The expression of programmed death-1 on CD4+ and CD8+ T lymphocytes in patients with type 2 diabetes and severe sepsis. PLoS One.

[38] Alhabeeb H, Baradwan S, Kord-Varkaneh H, Tan SC, Low TY, Alomar O (2021). Association between body mass index and urinary tract infection: a systematic review and meta-analysis of observational cohort studies. Eat Weight Disord: EWD.

[39] Liu J, Zhou C, Gao W, Huang H, Jiang X, Zhang D (2020). Does preoperative urine culture still play a role in predicting post-PCNL SIRS? A retrospective cohort study. Urolithiasis.

[40] Walton-Diaz A, Vinay JI, Barahona J, Daels P, González M, Hidalgo JP (2017). Concordance of renal stone culture: PMUC, RPUC, RSC and post-PCNL sepsis-a non-randomized prospective observation cohort study. Int Urol Nephrol.

[41] Liu M, Chen J, Gao M, Zeng H, Cui Y, Zhu Z (2021). Preoperative midstream urine cultures vs renal pelvic urine culture or stone culture in predicting systemic inflammatory response syndrome and urosepsis after percutaneous nephrolithotomy: a systematic review and meta-analysis. J Endourol.

[42] Mariappan P, Loong CW (2004). Midstream urine culture and sensitivity test is a poor predictor of infected urine proximal to the obstructing ureteral stone or infected stones: a prospective clinical study. J Urol.

[43] Ruan S, Chen Z, Zhu Z, Zeng H, Chen J, Chen H (2021). Value of preoperative urine white blood cell and nitrite in predicting postoperative infection following percutaneous nephrolithotomy: a meta-analysis. Transl Androl Urol.

[44] Flannigan R, Choy WH, Chew B, Lange D (2014). Renal struvite stones–pathogenesis, microbiology, and management strategies. Nat Rev Urol.

[45] Nevo A, Shahait M, Shah A, Jackman S, Averch T (2019). Defining a clinically significant struvite stone: a non-randomized retrospective study. Int Urol Nephrol.

[46] McAleer IM, Kaplan GW, Bradley JS, Carroll SF, Griffith DP (2003). Endotoxin content in renal calculi. J Urol.

[47] Motomura T, Shirabe K, Mano Y, Muto J, Toshima T, Umemoto Y (2013). Neutrophil-lymphocyte ratio reflects hepatocellular carcinoma recurrence after liver transplantation via inflammatory microenvironment. J Hepatol.

[48] Azevedo AS, Almeida C, Melo LF, Azevedo NF (2017). Impact of polymicrobial biofilms in catheter-associated urinary tract infections. Crit Rev Microbiol.

[49] Shander A, Lobel GP, Javidroozi M (2016). Transfusion practices and infectious risks. Expert Rev Hematol.

[50] Sihler KC, Napolitano LM (2010). Complications of massive transfusion. Chest.

[51] Aron M, Goel R, Gupta NP, Seth A (2005). Incidental detection of purulent fluid in kidney at percutaneous nephrolithotomy for branched renal calculi. J Endourol.

[52] Zhong W, Zeng G, Wu K, Li X, Chen W, Yang H (2008). Does a smaller tract in percutaneous nephrolithotomy contribute to high renal pelvic pressure and postoperative fever?. J Endourol.

[53] Degirmenci T, Bozkurt IH, Celik S, Yarimoglu S, Basmaci I, Sefik E (2019). Does leaving residual fragments after percutaneous nephrolithotomy in patients with positive stone culture and/or renal pelvic urine culture increase the risk of infectious complications?. Urolithiasis.

